# The length of a lantibiotic hinge region has profound influence on antimicrobial activity and host specificity

**DOI:** 10.3389/fmicb.2015.00011

**Published:** 2015-01-29

**Authors:** Liang Zhou, Auke J. van Heel, Oscar P. Kuipers

**Affiliations:** Department of Molecular Genetics, Groningen Biomolecular Sciences and Biotechnology Institute, University of GroningenGroningen, Netherlands

**Keywords:** lantibiotics, nisin, hinge region, membrane, diffusion

## Abstract

Lantibiotics are ribosomally synthesized (methyl)lanthionine containing peptides which can efficiently inhibit the growth of Gram-positive bacteria. As lantibiotics kill bacteria efficiently and resistance to them is difficult to be obtained, they have the potential to be used in many applications, e.g., in pharmaceutical industry or food industry. Nisin can inhibit the growth of Gram-positive bacteria by binding to lipid II and by making pores in their membrane. The C-terminal part of nisin is known to play an important role during translocation over the membrane and forming pore complexes. However, as the thickness of bacterial membranes varies between different species and environmental conditions, this property could have an influence on the pore forming activity of nisin. To investigate this, the so-called “hinge region” of nisin (residues NMK) was engineered to vary from one to six amino acid residues and specific activity against different indicators was compared. Antimicrobial activity in liquid culture assays showed that wild type nisin is most active, while truncation of the hinge region dramatically reduced the activity of the peptide. However, one or two amino acids extensions showed only slightly reduced activity against most indicator strains. Notably, some variants (+2, +1, −1, −2) exhibited higher antimicrobial activity than nisin in agar well diffusion assays against *Lactococcus lactis* MG1363, *Listeria monocytogenes*, *Enterococcus faecalis* VE14089, *Bacillus sporothermodurans* IC4 and *Bacillus cereus* 4153 at certain temperatures.

## Introduction

**Figure 1:**
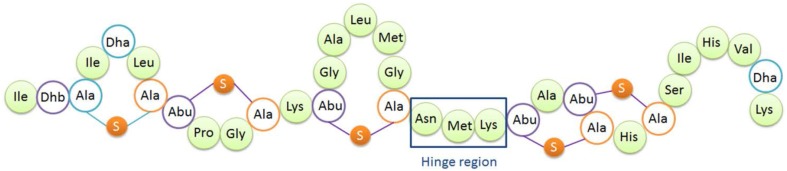
**Structure of nisin A**. Dha, dehydroalanine; Dhb, dehydrobutyrine; Ala-S-Ala, lanthionine; Abu-S-Ala, β-methyllanthionine. The hinge region (Asn-Met-Lys) is indicated.

The increasing occurrence of multi-drug resistance (Davies and Davies, 2010) has made the development of new antibiotics a priority. Lantibiotics (lanthionine-containing antibiotics) display strong activity against Gram-positive pathogens and can become a valuable addition to traditional antibiotics (van Heel et al., 2011). Nisin (Figure 1), is the prototype lantibiotic from *Lactococcus lactis* and it is active against Gram-positive bacteria at the nanomolar range. The biosynthesis of nisin involves ribosomal peptide synthesis, dehydration of serine and threonine residues, cyclization via sulfhydryl addition of cysteine to a dehydrated residue, transportation of the precursor peptide and proteolytic activation (Lubelski et al., 2008b). After ribosomal synthesis, nisin has a leader peptide part in front of the core peptide and is called prenisin. The leader peptide guides prenisin to NisB that dehydrates serines and threonines to form dehydroalanines (Dha) and dehydrobutyrines (Dhb). NisC can couple cysteines to dehydrated residues by an addition reaction, resulting in five (methyl)lanthionine rings in the structure of nisin. Subsequently, NisT transports the modified prenisin to the outside of the cell. Prenisin can be activated by cutting off the leader, a process that is catalyzed by the dedicated proteinase NisP.

Nisin has two inhibition mechanisms (Wiedemann et al., 2001; Breukink and de Kruijff, 2006): it binds to lipid II and causes pore formation. Lipid II is located in the membrane and plays an essential role in cell wall synthesis. Nisin can pass the peptidoglycan layer of Gram-positive bacteria and can bind to lipid II with the first two rings, which inhibits cell wall synthesis and bacterial growth. After binding, the C-terminal part of nisin will insert into the lipid bilayer, and subsequently a nisin -lipid II complex will be assembled (8:4) to form a pore complex in the membrane, eventually killing the bacterium (Hasper et al., 2004).

The so-called hinge region of nisin consisting of 3 amino acids (NMK) is located between the first three rings and the last two rings of nisin (see Figure 1). This region has been implicated to play an important role during insertion of the C-terminus of nisin into the membrane (Hasper et al., 2004). Mutagenesis of the hinge region has been studied intensively (Lubelski et al., 2008b; Ross and Vederas, 2011). The most recent research was conducted by Healy et al. (2013). Two mutants (AAA and SAA) were rationally designed based on random mutagenesis results, and the mutants showed enhanced activity against specific indicator strains such as *Lactococcus lactis* HP, *Streptococcus agalactiae* ATCC 13813, *Mycobacterium smegmatis* MC2155 and *Staphylococcus aureus* RF122.

So far, there has been no systematic study performed on the influence of the length of the hinge region on activity and spectrum. Nisin has to bend into the membrane and form a stable pore complex, and the membrane thickness of the bacteria might affect the activity of nisin. As we know, the thicknesses of bacterial membranes are affected by temperature (Cybulski et al., 2010) and different species can have different membrane thicknesses. To modulate the activity of nisin against bacteria with different membrane thicknesses, varying the length of the peptide could be effective. In the paper of Lubelski et al. (2008a), one to four alanines were added behind lysine 22 of nisin and the activities of these variants against *L.lactis* were tested, but the inhibition spectrum was not shown. In this current paper, the length of the nisin hinge region was varied from 1 to 6 amino acids. The variants were characterized by MS and activity assays on 10 different indicator strains. Profound effects of the length of the hinge region on the activity of the resulting peptides were demonstrated. This study represents a first trial on the rational design of the length of the hinge region assuming the difference in membrane thickness of targeted bacteria is important for effective membrane insertion.

## Materials and methods

### Bacterial strains and growth conditions

The bacterial strains used in this study are listed in Table 1. *L. lactis* strains were cultured in M17 broth supplemented with 0.5% (w/v) glucose (GM17) or GM17 agar for genetic manipulation or in minimal expression medium (MEM) for protein expression at 30°C (Rink et al., 2005). *E. faecalis*, *S. aureus*, *S. pneumonia*, and *L. monocytogenes* were grown in GM17 at 37°C or GM17 agar at different temperatures for agar diffusion assay. *M. luteus* was grown in Luria-Bertani broth shaken (200 rpm) at 30°C or LB agar at 20°C for agar diffusion assay. *Bacillus* strains were cultured in Brain Heart Infusion (BHI) shaken (200 rpm) or BHI agar at different temperatures for agar diffusion assay.

**Table 1:** **Strains and plasmids used in this study**.

**Strain or plasmids**	**Characteristics**	**References**
**STRAIN**		
*Lactococcus lactis* NZ9000	*nisRK*	Kuipers et al., 1997
**PLASMIDS**		
pIL3EryBTC	*nisBTC*, encoding nisin modification machinery, EryR^a^	van Heel et al., 2013
pNZ8048	Nisin inducible promoter in shuttle vector	de Ruyter et al., 1996
pNZnisA	*nisA*, encoding nisin, CmR^a^, inserted in pNZ8048	van Heel et al., 2013
pNZnisA H-2	*nisA*, encoding nisin, with methionine and lysine in the hinge region deleted	This study
pNZnisA H-1	*nisA*, encoding nisin, with methionine in the hinge region deleted	This study
pNZnisA H+1L	*nisA*, encoding nisin, with leucine inserted behind asparagine in the hinge region	This study
pNZnisA H+1V	*nisA*, encoding nisin, with valine inserted behind asparagine in the hinge region	This study
pNZnisA H+1I	*nisA*, encoding nisin, with isoleucine inserted behind asparagine in the hinge region	This study
pNZnisA H+2	*nisA*, encoding nisin, with isoleucine and valine inserted behind asparagine in the hinge region	This study
pNZnisA H+3	*nisA*, encoding nisin, with isoleucine, valine and leucine inserted behind asparagine in the hinge region	This study
Indicator strains		
*Lactococcus lactis* MG1363	Nisin sensitive indicator	Gasson, 1983
*Enterococcus faecalis* VE14089	Nisin sensitive indicator	Rigottier-Gois et al., 2011
*Staphylococcus aureus*	Nisin sensitive indicator	Lab collection
*Micrococcus luteus*	Nisin sensitive indicator	Lab collection
*Streptococcus pneumonia* R6	Nisin sensitive indicator	Lab collection
*Listeria monocytogenes*	Nisin sensitive indicator	Lab collection
*Bacillus sporothermodurans* IC4	Nisin sensitive indicator	TIFN collection
*Bacillus cereus* (L'29) 16	Nisin sensitive indicator	TIFN collection
*Bacillus cereus* 4147	Nisin sensitive indicator	TIFN collection
*Bacillus cereus* 4153	Nisin sensitive indicator	TIFN collection

a *EryR, erythromycin resistance; CmR, chloramphenicol resistance*.

### Molecular cloning

To obtain the hinge region variants, primers were designed to modify the DNA sequence of specific amino acids. With these primers (containing the modification on the 5′ end), the full plasmid was amplified. Phusion High-Fidelity DNA Polymerase (Thermo Scientific) was used to perform the PCR (Sambrook and Russell, 2001). PCR products were purified (Roche Switzerland) and ligated according to the manufacturer of the ligase (Thermo Scientific). The DNA sequence encoding the +3 variant was synthesized commercially (Life Technologies). Preparation of competent cells and transformation were performed as described previously (Holo and Nes, 1995).

### Expression, TCA precipitation and tricine SDS-PAGE

The expression strain *L. lactis* NZ9000 contained plasmids pIL3EryBTC and pNZnisA harboring different variants of the hinge region. Cells were cultured at 30°C first in GM17 medium with 4 μg/ml chloramphenicol and 4 μg/ml erythromycin until OD (600 nm) reached 0.7, then centrifuged and resuspended in MEM medium with 0.5% (w/v) glucose, 3 μg/ml chloramphenicol, 3 μg/ml erythromycin and 2 nM nisin to induce. After 3 h induction, the supernatant was harvested. The supernatant of small volume of fermentation (<10 ml) was concentrated by TCA precipitation (Sambrook and Russell, 2001) and the concentrated peptides were loaded on Tricine SDS-PAGE gel (Schägger, 2006) to check the amount and purity.

### Purification, characterization and quantification

>100 microgram of prenisin variants were purified by cation-ion exchange, desalting and freeze-drying (van Heel et al., 2013). The freeze dried peptides were dissolved and cut by trypsin overnight at 37°C in 50 mM Tris-HCl (pH 6.8) to separate the leader and core peptide. The digested product was further purified by HPLC (Agilent 1260 Infinity LC) equipped with a semi-preparative C12 column (Phenomenex 250 × 10 mm). The fractions were collected, tested for activity against *L. lactis* and analyzed with MALDI-TOF (van Heel et al., 2013). The active, fully dehydrated and pure fractions were freeze-dried. The freeze-dried peptides were dissolved with 0.05% acetic acid and first quantified by BCA (Thermo Scientific), using pure nisin as standard. The preliminary normalized peptides were further quantified with HPLC with an analytical C12 column (Phenomenex 250 × 4.60 mm). The concentration of the peptide was calculated by comparing the area of absorption peak at 226 nm with the area of different amount of pure nisin.

### Determination of the minimum inhibitory concentration (MIC)

The indicator strains were cultured until OD (600 nm) reached 0.5. Before MIC value test, the culture was diluted 1000 times to make the concentration of cells around 5 × 10^5^ per milliliter. The test was performed using a temperature controlled plate reader [Tecan200 (Tecan Group AG)] and 96 well plate (Greiner Bio-one). 180 μl indicator strain culture mixed with 20 μl hinge region analogs with final concentration from 0.031 μg/ml–32 μg/ml was incubated at 30°C or 37°C for 18 h. OD (600 nm) was checked every 30 min. For aerobic strains, 2 min of shaking was performed before every check. The concentration of peptide without observed growth of indicator strains was considered as the MIC value.

### Agar well diffusion assay

For the agar well diffusion assay, the same cultures as in the MIC value test were used and diluted 200 times with solid media. Identical sizes of wells (7.5 mm diameter) were made and 2 μg of hinge region analogs dissolved in 20 μl 0.05% acetic acid were added. The plates were incubated at different temperatures for at least 1 day until apparent halos appeared. For activity test at low temperature, the indicators were diluted in the same way but poured into smaller plates (52 mm diameter). The plates were incubated at 4°C overnight before the peptides were added. *B. sporothermodurans* IC4 and *B. cereus* 4153 were first incubated with the peptides at 4°C for 2 weeks then moved to 30°C and incubated overnight. The diameters of the halos were measured.

## Results

### The hinge region was varied from one to six amino acids

The hinge region is a positively charged and flexible part of nisin. According to previous mutagenesis results, incorporating negatively charged amino acids into the hinge region will greatly reduce the activity, while small, polar, hydrophobic or positively charged amino acids incorporation did not reduce the activity dramatically (Ross and Vederas, 2011). Therefore for this research, the nonpolar and hydrophobic amino acids valine, leucine and isoleucine were chosen to elongate the hinge region and inserted between the asparagine residue and methionine residue. In the two amino acids truncation variant, methionine and lysine were deleted. And in the one amino acid truncation variant, methionine was deleted. Table 2 shows the sequence of the hinge-region variants.

**Table 2:** **Sequence of the hinge region variants**.

**Name**	**Hinge region**
−2	N
−1	NK
Wild type	NMK
+1	NLMK
	NVMK
	NIMK
+2	NIVMK
+3	NIVLMK

### The hinge region variants show different extracellular expression levels

**Figure 2:**
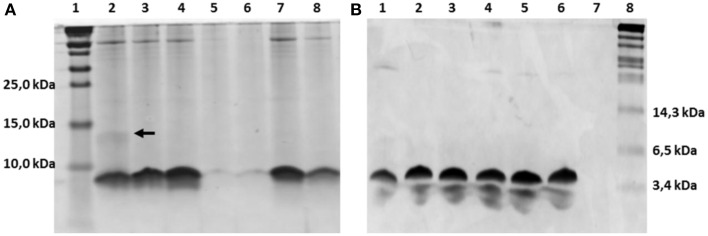
**Comassie stained tricine SDS-PAGE gel**. **(A)** TCA precipitated hinge region analogs. 1.8 ml supernatant was concentrated to 100 μl solution by TCA precipitation and 15 μl was loaded on the gel. Lane 1, protein marker (Thermo Scientific), the molecular weight are indicated on the left, from low to high are 10kDa, 15kDa and 25kDa; Lane 2–8, hinge region analogs. Lane 2, −2; Lane 3, −1; Lane 4, wild type; Lane 5, +1(valine); Lane 6, +1(leucine); Lane 7, +2; Lane 8, +3. The dimer of the −2 variant is indicated by an arrow. **(B)** Purified hinge region analogs (3 μg peptides were loaded per well). From left to right are −2 (lane 1), −1 (lane 2), wild type (lane 3), +1 (leucine) (lane 4), +2 (lane 5), +3 (lane 6) and the protein marker (Biolabs) (lane 8). The molecular weight of the marker are indicated, from low to high 3.4kDa, 6.5kDa, and 14.3kDa.

The nisin-inducible two plasmids expression and modification system (Kluskens et al., 2005) was used to express nisin and the hinge region variants. In Figure 2A the production of different hinge region variants is compared by loading TCA precipitated supernatants on a tricine SDS-PAGE gel. From lane 4 we can see that nisin is produced at a good amount. The −2 (lane 2),−1 (lane3) and +2 (lane 7) variants showed an almost equal level of production compared to nisin. The +3 (lane 8) variant showed about half the amount of production. However, the +1 (lane 5 and lane 6) variants showed about 100 times lower production than wild type. And this deficiency did not change when the inserted amino acid was changed from isoleucine (data not shown) to valine or leucine. As the leucine insertion analog showed a slightly improved production level, in the following experiments, the +1L was used.

### Variation of the hinge region does not change the degree of dehydration of the peptide except for the −2 peptide

The dehydrated residues and the (methyl)lanthionine rings are very important for the activity of lantibiotics. And these modifications are catalyzed by NisB and NisC (Lubelski et al., 2008b). However, varying the length of the hinge region can change the distance between the modifiable residues and the leader and affect the behavior of NisB and NisC. To make sure the variants were fully modified, MALDI-TOF was used to assess the dehydration extent of the peptides (Table 3). The mass of the TCA precipitated peptides indicated that the −2 peptide was dehydrated 7 times and other analogs were fully dehydrated (−8 H_2_O). Also the −2 analog has more tendency to form a dimer (see arrow in Figure 2A), and this has been confirmed by western blot (data not shown). However, by large scale purification, the 8 times dehydrated −2 variant was also obtained through isolation by HPLC (Table 3). Figure 2B shows the purified peptides.

**Table 3:** **Molecular mass of hinge region analogs detected by MALDI-TOF**.

**Hinge region analogs**	**Number of dehydration**	**TCA precipitated prepeptides**		**Peptides after large scale purification (without leader)**	
		**Predicted mass(Da)**	**Observed mass(Da)**	**Predicted mass(Da)**	**Observed mass(Da)**
−2	8	5427.9		3095.7	3093.1
	7	5445.9	5447.5	3113.7	
−1	8	5556.1	5560.1	3223.9	3224.2
WT	8	5687.3	5686.9	3355.1	3353.5
+1L	8	5800.7	5800.7	3468.5	3467.4
+2	8	5899.6	5899.2	3567.4	3564.4
+3	8	6012.8	6013.8	3680.6	3681.0

### Varying the length of the hinge region changes the antimicrobial activity of nisin, in a target-specific way

The MIC values of 10 different indicator strains were assessed for the hinge region mutants and wild type. Literature indicates that *Staphylococcus* and *Micrococcus* commonly have thinner membranes, while *Streptococcus* and *Lactococcus lactis* have a thicker membrane (Bierbaum and Sahl, 2009). In Table 4, the strain-specific activities of hinge region variants are shown. To compare activity of all analogs, the residual activity of analogs of nisin were calculated. From the ratio, we can see wild type nisin has the best activity against all indicators. The −2 variant lost most of the activity. One amino acid deletion was also detrimental for the activity, but against *S. pneumoniae* and *B. sporothermodurans*, which grow slowly, the −1 variant showed less reduced activity than other analogs. One or two amino acids elongation changed the activity only modestly. Against *Enterococcus faecalis*, the +1 showed better activity than +2, while against *Listeria monocytogenes, Bacillus cereus* 4147 and 4153, the +2 variant showed relatively higher activity. Activity against *Staphylococcus aureus* was dramatically reduced when changing the length of the hinge region.

**Table 4:** **Minimum inhibitory concentration (MIC) of the hinge-region variants against representative Gram-positive strains, determined in liquid culture**.

**Indicators**	**MIC value (μg/ml)**						**Residual activity of variants compared to nisin (%)^a^**					
	
	**−2**	**−1**	**WT**	**+1**	**+2**	**+3**	**−2**	**−1**	**WT**	**+1**	**+2**	**+3**
*Enterococcus faecalis* VE14089	>32	>32	1.7	4	5.3	>8	<6	<6	100	37	30	<21
*Listeria monocytogenes*	>8	>8	2	8	4	>8	<25	<25	100	25	50	<25
*Bacillus cereus* 4147	>32	>32	4	16	8	16	<13	<13	100	25	50	25
*Bacillus cereus* 4153	>32	>32	4	16	8	32	<13	<13	100	25	50	13
*Lactococcus lactis* MG1363	0.5	1	0.03	0.125	0.125	0.25	6	3	100	25	25	13
*Bacillus cereus* (L′29) 16	>32	>32	4	16	16	32	<13	<13	100	25	25	13
*Micrococcus luteus*	>8	>8	2	8	8	8	<25	<25	100	25	25	25
*Streptococcus pneumoniae* R6	8	4	1	6	4	8	13	25	100	17	25	13
*Bacillus sporothermodurans* IC4	3	0.75	0.375	1	1	2	13	50	100	33	33	17
*Staphylococcus aureus*	>16	16	0.5	8	>8	>16	<3	3	100	6	<6	<3

a *The percentage of residual activity was calculated by dividing the MIC value of nisin by that of the analogs*.

### Activity of hinge region analogues in solid media

The antimicrobial activity of the hinge region analogs in solid media were tested by an agar well diffusion assay. The diameters of the halos were measured and the residual activity of the analogs were estimated according to the formula of the relationship between the concentration of nisin and the sizes of halos. We made use of the fact that the size of the halo is directly related to the log of nisin concentration. Of course this is an estimate of activity of the variants because any changes in diffusion of the variant bacteriocin or growth rates of indicator bacteria during the assay could also affect the size of the halo. (Table 5 and Supplementary Figure 1). The plates were shown in supplementary data (Supplementary Figures 2–8). As we can see, nisin did not always show the best activity. More specifically, against *L. lactis*, *L. monocytogenes* and *E. faecalis*, the +2 variant showed a higher or equal activity compared to nisin. And the +1 variant, which is not as efficient as the +2, also displayed almost equal or better activity than nisin. In contrast to the MIC value test in liquid culture, the −2 variant showed large halos in the plate activity test against these three strains. And against *L. lactis* at 20°C, the inhibition zone was larger than nisin. The −1 variant showed about 30% residual activity against *L. lactis* and *L. monocytogenes* at all tested temperatures. However, against *E. faecalis*, the −1 variant showed an increased activity when the temperature goes higher (from 21 to 140%). Notably, against *B. sporothermodurans*, the −1 variant showed an apparently larger halo than nisin, which is in accordance with the MIC value test. And the +2 variant also showed higher activity than nisin. The *Bacillus cereus* are difficult to be inhibited by nisin, and the +2 variant displayed either equal or higher plate activity than nisin at 30°C (Supplementary Figure 8). The *M. luteus* grows quite fast at room temperature and only the +2 variant retained 55% of the activity of nisin. Against *S. aureus*, the analogs displayed dramatically reduced activity. And in this case, the +1 variant displayed relatively higher activity than the +2 variant. In all the cases, the +3 variant displayed dramatically reduced activity.

**Table 5:**
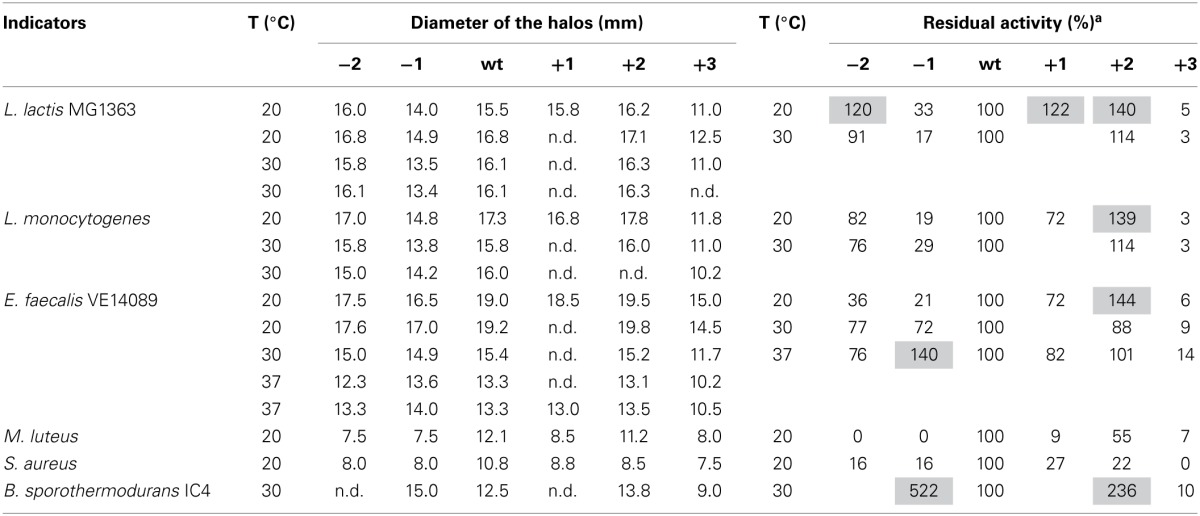
**Activities of the hinge region analogs in agar well diffusion assay**.

*^a^The residual activity is estimated according to a standard curve of various nisin concentrations plotted against diameter (halo diameter is directly related to log of nisin concentration). Improvements in residual activity are indicated in gray. n.d.: not determined*.

### The inhibition capability of nisin and +2 variant at low temperature

**Figure 3:**
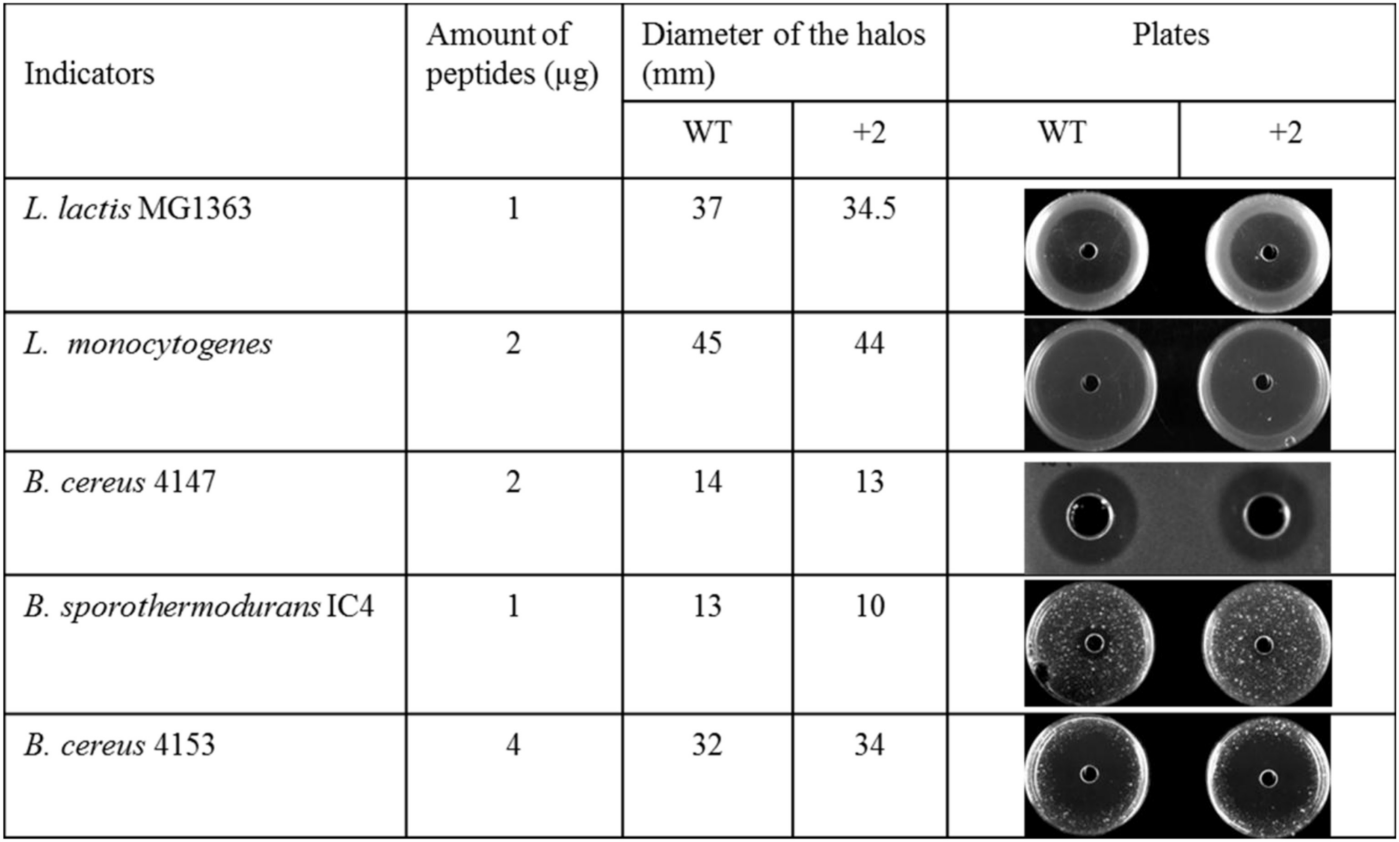
**Activity of nisin and +2 variant in solid media at low temperature**.

Some food spoilage bacteria e.g., *Bacillus cereus* and *Listeria monocytogenes*, can grow at low temperature. In this research, several kinds of bacteria were chosen to test their sensitivity to nisin and the hinge region analog +2 at low temperatures (Figure 3). The *L. lactis* and *L. monocytogenes* grow well at 4°C. After 1 week, big halos can be seen in the plates. Against *L. lactis*, nisin showed an apparently larger halo than the +2 variant, which are different from the results at higher temperature. Against *L. monocytogenes*, both wild type and the +2 displayed high activity but the wild type can inhibit the growth of bacteria a bit more efficiently than +2. *B. cereus* 4147 cannot grow at 4°C but grow well at 12°C. However, the +2 variant also showed lower activity than nisin at this temperature. As the *Bacillus* did not show apparent growth after incubated with the peptides at 4°C for 2 weeks, the plates were moved to 30°C and incubated overnight. In this case, the *Bacillus* grow fast and various sizes of colonies were shown. As the figure shows, the wild type displayed significantly higher activity than +2 against *B. sporothermodurans*, while against *B. cereus* 4153, the +2 variant showed a modestly larger halo than nisin.

## Discussion

Nisin inhibits the growth of Gram-positive bacteria by binding to lipid II, inserting into the membrane and forming stable pores in the membrane. However, the thicknesses of the membrane vary between different species and growth conditions. This difference can affect the activity of the peptide. For example, some lantibiotics with shorter length, e.g., epidermin (Bonelli et al., 2006), gallidermin (Bonelli et al., 2006), mutacin 1140 (Smith et al., 2008) and bovicin HC5 (Paiva et al., 2011), display lipid II binding activity but only form pores when the targeted bacteria or liposomes have a thinner membrane (not exceeding 40 Å). These results indicate that the length of the lantibiotics, especially at the C-terminal part, could be optimized to efficiently inhibit growth of bacteria with different membrane thicknesses. In this paper, the length of the hinge region of nisin was varied to make the peptides have different lengths of C-terminus.

Three similar amino acids (I, V, L) were used to elongate the hinge region at the same position (between N20 and M21). Notably, all +1 variants showed an extremely low production level. Since the dehydration extent of the +1 variant was normal and since this peptide showed good activity, the modification machinery seems to work properly. To understand the reasons for the low production level, additional different amino acids could be inserted at different positions of the hinge region. Especially since previously it was shown that inserting an alanine at the end of the hinge region (NMKA) did not affect the production level (Lubelski et al., 2008a).

Specific activity of the variants was tested against different indicators. As the hinge region connects the first three rings and the intertwined rings of nisin, deletion of one or two amino acids will reduce the flexibility of the peptide and the pore formation activity will be weakened or abolished. The results show that these truncations are detrimental for activity against all indicators, which proves that the flexibility of the hinge region is important for the activity of nisin. This has also been shown before by proline-proline containing variants (Kuipers et al., 1996). Elongation of the hinge region can potentially enhance the activity of peptides against those bacteria with thicker membrane. In this paper, one or two amino acids extensions only modestly reduced the activity. This indicated that a slightly longer hinge region did not greatly affect the function of nisin and the pore formation activity could still be performed. The relatively lower activity is probably because the addition of leucine (+1), or isoleucine and valine (+2) changed the amphipathicity of the C-terminus of nisin, due to which the peptide cannot perform the pore formation efficiently. If the added amino acids and position of the insertion can be randomly changed, probably higher activity can be obtained. According to the literatures, some point mutants in the hinge region showed enhanced or modestly reduced activity, e.g., N20 (P,K), M21(V,K,G), K22(T,S) (Yuan et al., 2004; Field et al., 2008) and the combinations of AAK, NAI, SLS, AAA and SAA have been shown to have high activity (Healy et al., 2013). These residues can be good candidates to elongate the hinge region. Furthermore, the elongated variants showed 16 times or more reduced activity against *Staphylococcus aureus*, which indicates significant strain specificity of the +1 and +2 variants. This bacterium was described to have a thinner membrane (Bonelli et al., 2006), but whether longer peptides have reduced activities against bacteria with thinner membranes needs to be further proven.

As it can be seen, the peptides showed different inhibition capability in the solid media compared to the MIC value test. This can be related to the growth differences of the strain and diffusion rate of the peptides. Against some indicators (e.g., *M. luteus*, *S. aureus*), the hinge region analogs did not show any advantages compared to nisin, and nisin showed much better activity than the +2 variant at low temperatures, which indicate that after a long time of evolution, nisin has more advantages than the analogs in most of the conditions.

Increasing the activity of nisin is very hard, because it is already evolutionary optimized against its natural targets. But engineering of nisin can change the properties of the peptide and the analogs can be applied against specific targets. To be used as a valuable antimicrobial reagent, the production level is very important. The +1 variant is not suited in this respect although the activity is relatively high. The −2 variant is also not a good candidate for application because of the incomplete dehydration. The +2 variant showed good properties and can be specifically used to inhibit growth of *L. lactis* MG1363 and *L. monocytogenes* at room temperature or higher, *B. cereus* 4153 in refrigerator or at 30°C in solid media. Both the +2 and −1 variant can be used to inhibit growth of *B. sporothermodurans* IC4 at 30°C in solid media. It has been shown that the thickness of the lipid bilayer is affected by temperature and the membrane will become thinner as the temperature goes up (Szekely et al., 2011). In this research, the activities of hinge region analogs against *E. faecalis* showed temperature dependence in the agar well diffusion assay. As the temperature goes up, the peptide with a shorter hinge region (−1 variant) displayed higher activity. So the −1 variant can be applied at 37°C in solid media against *E. faecalis*. At room temperature, this bacterium grows more slowly, and then the +2 variant can inhibit its growth more efficiently. This study shows that by engineering the length of the hinge region of nisin, variants with a changed host range can be obtained, but that the effects are very host range- and temperature dependent. Moreover, some variants display better activity in a solid test medium, while being worse in liquid culture. Thus, the choice of wild-type or variant nisin will depend on the specific application, temperature and matrix.

### Conflict of interest statement

The authors declare that the research was conducted in the absence of any commercial or financial relationships that could be construed as a potential conflict of interest.
