# Supplementary material for: The length of a lantibiotic hinge region has profound influence on antimicrobial activity and host specificity
Source: Front Microbiol. 2015 Jan 29;6:11. doi: 10.3389/fmicb.2015.00011 (PMC4310329; doi:10.3389/fmicb.2015.00011)
Supplement: Supplementary file 1 [file Image1.PDF]

Supplementary Material

The length of a lantibiotic hinge region has profound influence on antimicrobial activity and host specificity

Liang Zhou[1], Auke J. van Heel[1], Oscar P. Kuipers[1]*

[1]Department of Molecular Genetics, Groningen Biomolecular Sciences and Biotechnology Institute, University of Groningen, Groningen, the Netherlands
*** Correspondence:** Oscar P. Kuipers, Department of Molecular Genetics, Groningen Biomolecular Sciences and Biotechnology Institute, University of Groningen, Groningen, Nijenborgh 7, 9747 AG, The Netherlands.
o.p.kuipers@rug.nl

1. Supplementary data

To evaluate the activity of nisin on the agar plate, an agar well diffusion assay with gradient of nisin against *Lactococcus lactis* (with two plasmids) was performed (**Supplementary Figure 1A**). The diameter of the halos were measured (**Supplementary Figure 1B**). The data shows that the log10 of the concentration of nisin is in liner relationship with diameter of the halos (**Supplementary Figure 1C**). According to the formula (1), the relative activity of the peptides compared to nisin can be calculated by the formula (2).

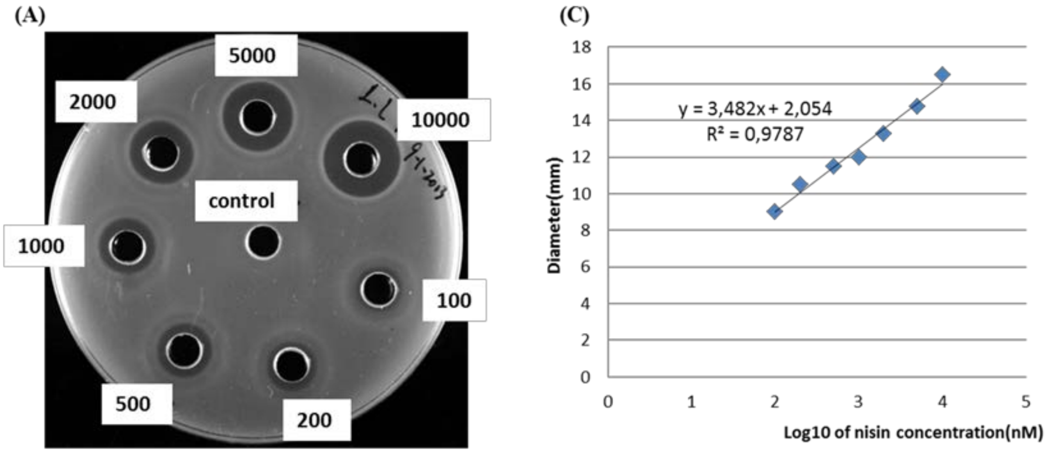

(B)

Concentration(nM)	100	200	500	1000	2000	5000	10000
log10	2,0	2,3	2,7	3,0	3,3	3,7	4,0
Diameter(mm)	9,0	10,5	11,5	12,0	13,3	14,8	16,5

Supplementary Figure 1. Relationship between concentration of nisin and diameter of the halos. (A) Agar well diffusion assay. Gradient concentration of nisin were made and dissolved in 30µl 0.05% acetic acid. *Lactococcus lactis* (with two plasmids) was cultured overnight and diluted 250 times with GM17 agar before added to the plate. The final concentration of the cells was about 2×10^6 cells/milliliter. The plate was incubated for 1 day. Concentrations of nisin were labeled. **(B) log10 of the concentrations and diameters of the halo. (C) A linear curve was made between the diameters of the halo and log10s of the concentration of nisin.**

$$d = 3{,}482\ log_{10}\ C + 2{,}054 \qquad (1)$$

$$Residual\ activity = 10^{\frac{d - d_{\circ}}{3{,}482}} \qquad (2)$$

d = diameter of the halo
C = concentration of nisin
$d_{\circ}$ = diameter of the halo of wild type

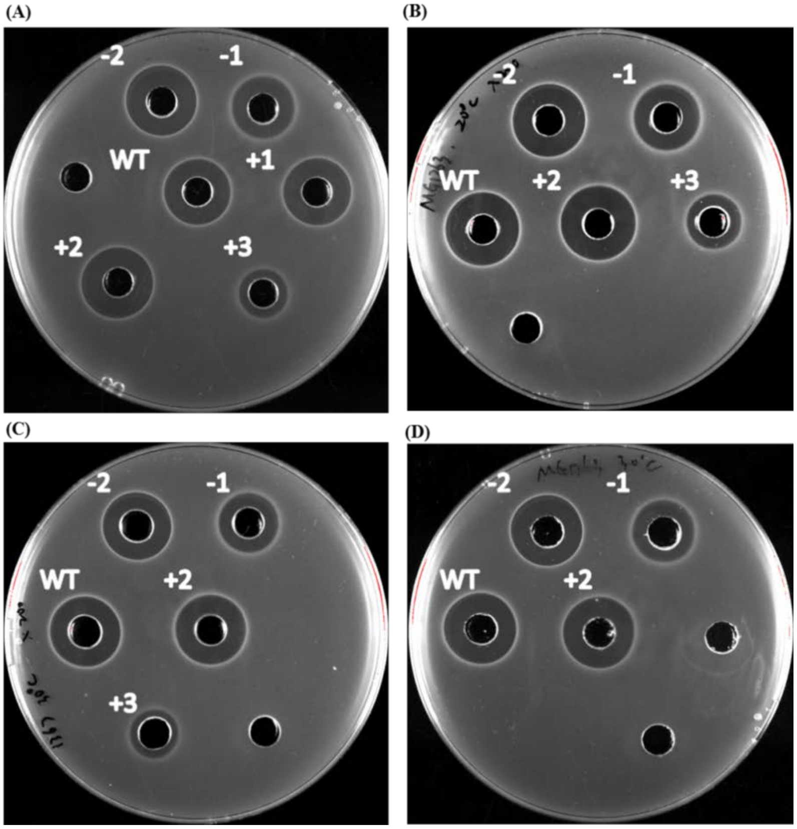

Supplementary Figure 2. Agar well diffusion assays of hinge region analogues against *Lactococcus lactis* MG1363. 2 µg of peptides were added per well. The plates were incubated either at 20 °C for 3 days (A) and (B), or at 30°C for 1 day (C) and (D).

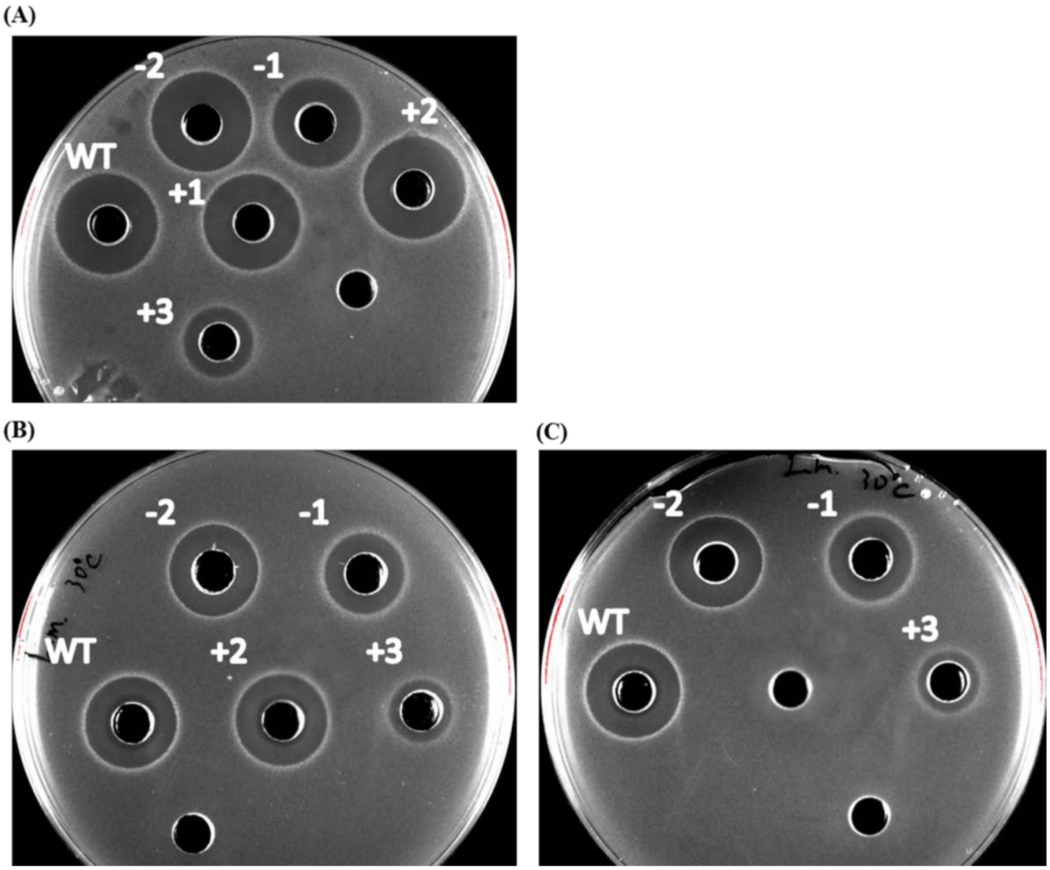

Supplementary Figure 3. Agar well diffusion assays of hinge region analogues against *Listeria monocytogenes*. 2 µg of peptides were added per well. The plates were incubated either at 20 °C for 3 days(A), or at 30°C for 1 day (B) and (C).

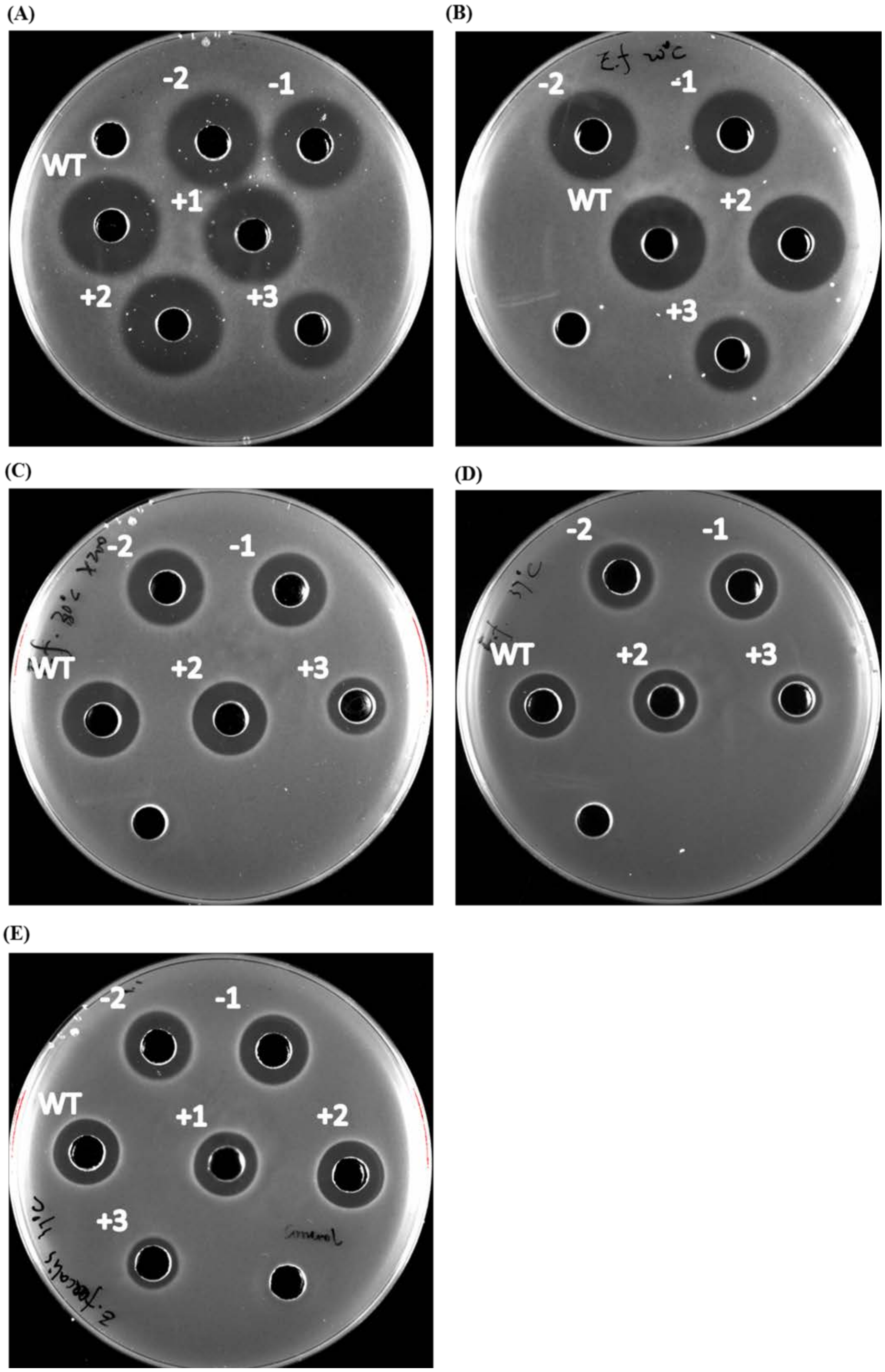

Supplementary Figure 4. Agar well diffusion assays of hinge region analogues against *Enterococcus faecalis* VE14089. 2 µg of peptides were added per well. The plates were incubated either at 20 °C for 3 days (A), or 2 days (B), or at 30°C for 1 day(C), or at 37°C for 1 day (D) and (E).

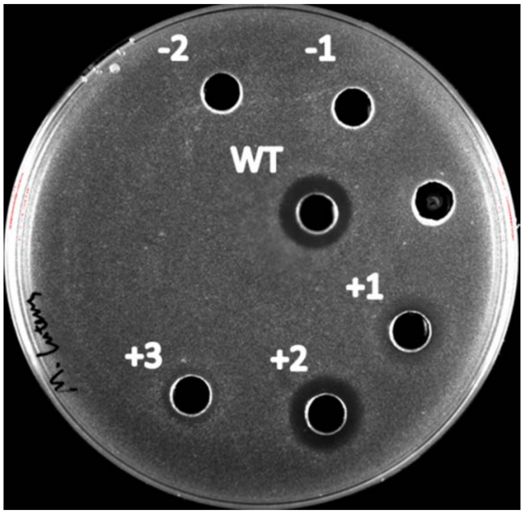

Supplementary Figure 5. Agar well diffusion assays of hinge region analogues against *Micrococcus luteus*. 2 µg of peptides were added per well. The plates were incubated at 20 °C for 1 day.

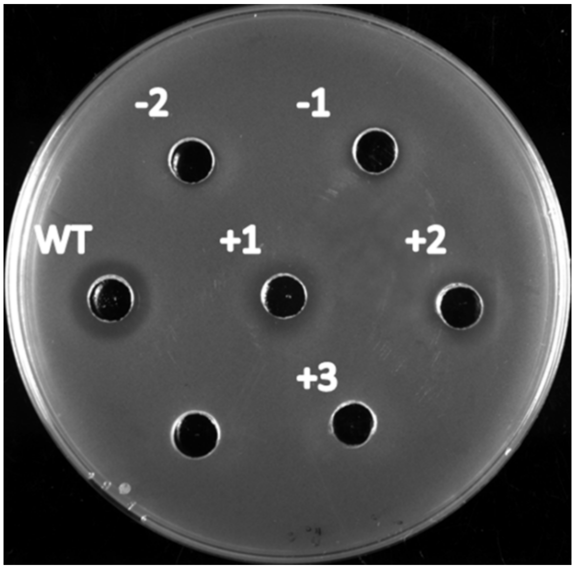

Supplementary Figure 6. Agar well diffusion assays of hinge region analogues against *Staphylococcus aureus*. 2 µg of peptides were added per well. The plates were incubated at 20 °C for 3 days.

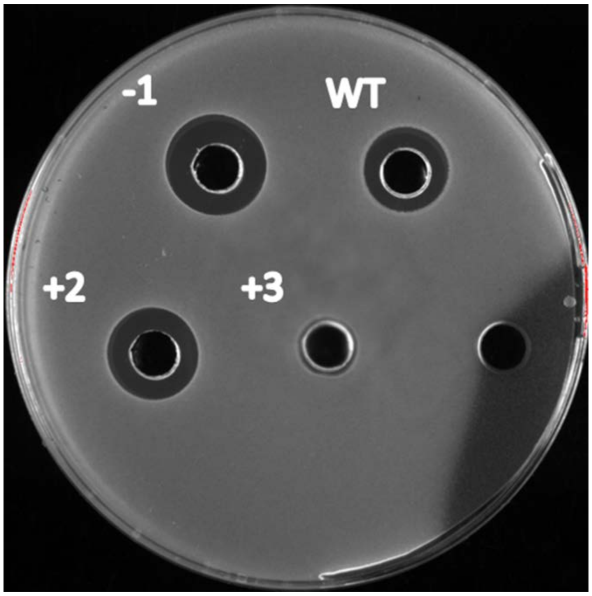

Supplementary Figure 7. Agar well diffusion assays of hinge region analogues against *Bacillus sporothermodurans* IC4. 2 µg of peptides were added per well. The plates were incubated at 30 °C for 2 days.

Strains	Diameter of the halo(mm)		Plate	
	WT	+2	WT	+2
B. cereus (L' 29) 16	10.5	10,5	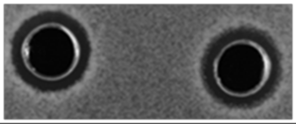	
B. cereus 4147	11.3	11.3	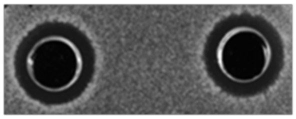	
B. cereus 4153	11.6	12.0	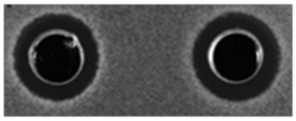	
B. sporothermodurans IC4	15.4	16.6	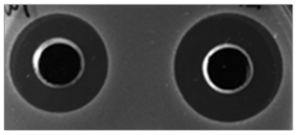	

Supplementary Figure 8. Agar well diffusion assays of hinge region analogues against 4 kinds of *Bacillus* strains. 5µl overnight culture was mixed with 5ml BHI agar and poured into the plate. Either 5µg nisin or the +2 analogue, dissolved in 50µl 0.05% acetic acid, was loaded. The plates were incubated at 30°C for 18 hours.
